# Assessing the use of hospital staff influenza-like absence (ILA) for enhancing hospital preparedness and national surveillance

**DOI:** 10.1186/s12879-015-0789-z

**Published:** 2015-03-01

**Authors:** Lydia N Drumright, Simon DW Frost, Alex J Elliot, Mike Catchpole, Richard G Pebody, Mark Atkins, John Harrison, Penny Parker, Alison H Holmes

**Affiliations:** Department of Medicine, University of Cambridge, Cambridge, UK; National Centre for Infection Prevention and Management and National Institute for Health Research Health Protection Research Unit in Healthcare Associated Infection and Antimicrobial Resistance Imperial College London, London, UK; Department of Veterinary Medicine, University of Cambridge, Cambridge, UK; Real-time Syndromic Surveillance Team, Public Health England, Birmingham, UK; Centre for Infectious Disease Surveillance and Control, Public Health England, London, UK; Department of Virology, Imperial College Healthcare NHS Trust, London, UK; Department of Occupational Health, Imperial College Healthcare NHS Trust, London, UK; Department of Human Resources, Imperial College Healthcare NHS Trust, London, UK; Department of Infection Prevention and Control, Imperial College Healthcare NHS Trust, London, UK

**Keywords:** Influenza, Syndromic surveillance, Healthcare workers, Emergency preparedness, Informatics, Epidemiology

## Abstract

**Background:**

Early warning and robust estimation of influenza burden are critical to inform hospital preparedness and operational, treatment, and vaccination policies. Methods to enhance influenza-like illness (ILI) surveillance are regularly reviewed. We investigated the use of hospital staff ‘influenza-like absences’ (hospital staff-ILA), i.e. absence attributed to colds and influenza, to improve capture of influenza dynamics and provide resilience for hospitals.

**Methods:**

Numbers and rates of hospital staff-ILA were compared to regional surveillance data on ILI primary-care presentations (15–64 years) and to counts of laboratory confirmed cases among hospitalised patients from April 2008 to April 2013 inclusive. Analyses were used to determine comparability of the ILI and hospital-ILA and how systems compared in early warning and estimating the burden of disease.

**Results:**

Among 20,021 reported hospital-ILA and 4661 community ILI cases, correlations in counts were high and consistency in illness measurements was observed. In time series analyses, both hospital-ILA and ILI showed similar timing of the seasonal component. Hospital-ILA data often commenced and peaked earlier than ILI according to a Bayesian prospective alarm algorithm. Hospital-ILA rates were more comparable to model-based estimates of ‘true’ influenza burden than ILI.

**Conclusions:**

Hospital-ILA appears to have the potential to be a robust, yet simple syndromic surveillance method that could be used to enhance estimates of disease burden and early warning, and assist with local hospital preparedness.

**Electronic supplementary material:**

The online version of this article (doi:10.1186/s12879-015-0789-z) contains supplementary material, which is available to authorized users.

## Background

Recognition that earlier detection of infectious diseases at the population level is critical for reducing morbidity and mortality has led to the extensive use of syndromic surveillance, i.e. monitoring a collection of symptoms purported to identify a particular condition. While the 2009 H1N1 influenza pandemic (pH1N1) demonstrated the critical value of such surveillance systems, it also highlighted inherent shortcomings [1]. The World Health Organization (WHO) review of influenza surveillance following pH1N1 underscored critical gaps in assessing influenza annually, including an inability for most countries to quantify burden of disease and distinguish intensity between seasons, and non-standardized, and therefore non-comparable, approaches nationally and internationally [2]. Similarly, problems related to managing a surge in patients with influenza in hospitals have been highlighted as a weakness in preparedness in Europe [3]. As timing and severity of influenza changes annually, delays in information reaching healthcare settings could result in a lack of readiness, especially related to hospital workforce staffing, with consequent risks of compromising patient safety and increased mortality [4,5].

Monitoring of primary care influenza-like illness (ILI) presentations is recommended by WHO as part of a minimum influenza surveillance strategy [2], and is used for early detection of influenza in the United Kingdom (UK) [6] and most other Western countries [2,7], and subsequent planning of health resources. In the UK, ILI surveillance data are principally collected by primary-care providers, and are supplemented by surveillance of severe cases [2,7,8], and other community reporting, e.g., medical helplines [6,9]. When modelled using multiple data sources, these systems provide a good estimate of seasonal influenza dynamics, but they are hindered by unavoidable presentation bias, limiting the ability to capture the full spectrum of disease across a population [10], and in particular the burden of influenza annually, which has been highlighted as a key influenza surveillance objective [2,3]. Additionally, whilst data are reported daily, even short delays in processing and disseminating data alongside varying seasonal patterns in different years can leave hospitals unprepared for staffing shortages, particularly if concentrated in certain departments.

Presentation bias is an important issue. In longitudinal household studies, 20-50% of those with influenza who experienced ILI visited a primary-care setting where healthcare visits were free at the point of care [11,12], and only 3% where there was a cost at the point of care [13]. While these studies may represent extreme ends of the spectrum in accessing care, they illustrate the wide difference in healthcare-seeking behaviours in different settings. Such differences were also observed in the UK during the pH1N1 introduction when the community was urged to seek healthcare if they experienced ILI symptoms, resulting in increased primary-care visits given the relatively low burden of disease. In the subsequent winter (2009/2010) a specialty triage telephone line was established, causing a reduction in primary-care visits relative to disease burden [1,11]. Additionally, ILI primary-care visits in the UK generally occur two or more days after onset of symptoms [14]. These patterns of behaviour reduce both the completeness and timeliness of detection of ILI, and delay warnings to healthcare systems. As these data come from healthcare presentation, they are unlikely to give hospitals very much lead time to prepare for a surge of patient presentations and they may not provide pre-emptive insight into patient-facing staff absences.

A number of innovative ILI sentinel sources have been explored to enhance early warning and estimation of the burden of disease, including pharmaceutical sales, emergency department visits, health helpline calls [15], social media [16-18], volunteer community self-report surveillance [19] and school absences [20,21]. The opportunities for syndromic surveillance are expanding with the advent of more sophisticated modern electronic databases and the use of ‘Big Data’ [22-24]. While these methods provide varying degrees of information on mild to severe respiratory illness in the community, those related to health-seeking suffer from presentation bias and we have yet to fully understand the validity of social media, such as increase in internet searches for the term influenza [25]. School absences, while promising, often contain all absences, without specification of reason, and are limited to term time and week days. Hospital staff illness absences, on the other hand, are recorded all year around and within the NHS provide data on absence reasons. They also have the added benefit of providing real-time information to hospitals about staffing levels that could support continuity of care.

We addressed the feasibility and efficiency of a novel syndromic surveillance method using hospital staff cold, cough, and influenza absences to extend local and national influenza surveillance, improve existing systems, and inform local hospital preparedness.

## Methods

We performed surveillance analyses on data from staff absences reported to Human Resources (HR) at the Imperial College Healthcare NHS Trust (ICHT), Royal College of General Practitioners (RCGP) ILI incidence data for the London Strategic Health Authority (SHA), and positive influenza test results from ICHT inpatients. The use of hospital staff data was approved by the UK National Research Ethics Committee, and all data were anonymized before analysis. Analyses were based on data aggregated into weekly counts, stripped of any personal identifiers.

### Sources of data and definitions

#### ICHT hospital staff influenza-like absence (hospital staff-ILA)

ICHT is one of the largest NHS hospital organisations in the UK, including five hospitals, nine satellite clinics, 1200 beds, and approximately 9500 staff. The reason and date of illness for all staff absences are recorded and entered into an electronic reporting system as standard practice at ICHT. Start dates of absences due to “cold”, “cough”, “influenza”, or any combination thereof were aggregated into weekly counts, over the period from week 14 in 2008 (ISO-8601 numbering, commencing 2008-03-31) to week 17 in 2013 (ending 2013-04-28); and monthly data on the total number of staff in post were used to calculate rates.

All staff from all hospital and satellite clinics were combined in the analyses. The vast majority of these staff were “patient-facing” e.g., clinical doctors and nurses, reception, etc.. Illness absence for each staff member was regarded as a single episode if the absence was for the same illness and on consecutive days, for example five days of absence due to influenza was counted as a single absence and attributed to the week of the first day of absence.

#### RCGP London Strategic Health Authority ILI (London-ILI)

The RCGP collect ILI data from a network of participating primary-care physicians who report on all patients attending their clinic who meet a standardised definition. RCGP ILI data from the London Strategic Health Authority (SHA) were collated, comprising of weekly counts by age group from week 14 in 2008 to week 17 in 2013. Corresponding population sizes, comprising of the number of patients registered at reporting practices, were used to obtain rates. To aid comparability with the staff absence data, we restricted analysis to cases between 15 and 64 years of age inclusive, as standard ‘adult’ age groupings start with 15–24 years.

Due to recent changes in the RCGP service provider’s database system for illness, the number of sentinel sites reporting temporarily decreased resulting in a reduction in the population size for which ILI was observed from approximately 106,000 patients to around 35,000 patients from the middle of 2012 for the London SHA.

#### Inpatient positive influenza test results (inpatient-PITR)

All cases of influenza, defined as having one or more positive tests for influenza, among patients in the three acute care hospitals within the ICHT were included, from week 14 in 2008 until week 17 in 2013. From March 2008 through June 2009 influenza A and B viruses were detected using direct immunofluorescence (Light Diagnostics, EMD Millipore Ltd., UK) and virus isolation in cell culture from nasopharyngeal aspirates (NPA) or bronchoalveolar lavage (BAL). From July 2009, influenza A and B viruses were detected using real-time polymerase chain reaction to detect influenza RNA (adapted from “CDC protocol of real time RT-PCR for swine influenza A (H1N1) 28/04/2009. CDC Ref. #I-007-05.”).

### Statistical analyses

Multiple quantitative approaches were used to assess the comparability of hospital staff-ILA to London-ILI. To investigate the overall correlation between hospital staff-ILA and London-ILA, we calculated the cross-correlation of log_10_ transformed time series for different time lags. We used a prospective outbreak detection algorithm to establish the timing of ‘alarms’, i.e. when the number of cases began to increase, to determine if hospital-ILA could provide earlier warning for hospital preparedness, and to compare the dynamics of ILA with ILI, particularly at low levels. We employed a Bayes subsystem [26], using reference values from the previous six weeks due to relatively short, heterogeneous time series, modified to take into account potentially fluctuating population sizes. An upper bound for the number of cases is calculated based on this distribution (we used the 0.95 quantile), and an alarm is raised if the number of cases is the same or exceeds this upper bound. The resulting threshold from this approach varies over time, such that multiple ‘alarms’ may be triggered in a given season. We also calculated epidemic threshold for each season using the Moving Epidemic Method [27], which identifies the epidemic period, and hence generates a single ‘alarm’ per season. To investigate the comparability of seasonal patterns in more detail, we decomposed the log_10_ transformed time series into seasonal, trend, and irregular components using loess smoothing (known as an ‘STL’ approach). To determine the comparability of estimates of disease burden, we compared hospital staff-ILA and London-ILI with predictions of an age-structured epidemiological model developed by Birrell et al. [1]. Estimates of numbers of influenza symptomatic cases and all infections for the London SHA for weeks 19–52 for 15–64 year olds, in the form of posterior medians and 95% credible intervals, were converted into rates per 100,000, assuming a constant population size over the time period. All analyses were performed in R v. 3.1.0 [28], using the surveillance (v1.7.0) and mem (v1.4) libraries [29,30].

## Results

Over the study period, there were 20,021 reported hospital staff-ILA at ICHT (with a median of 9577 staff monthly), 1197 inpatient-PITR, and 4661 community London-ILI cases (median population size 103,666).

### Similarities in surveillance patterns

**Figure 1:**
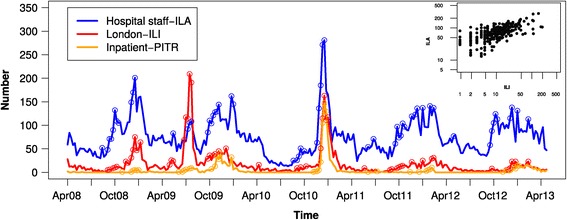
**Weekly counts of hospital staff-ILA (blue), hospital inpatient-PITR (orange), and London-ILI in the community (red) from April 2008 to April 2013 and prospective alarms for elevated counts (circles) using a Bayesian subsystem algorithm, using the previous six weeks as the reference for prediction.** Data plotted by counts rather than rates for clarity. (inset) Scatterplot of hospital staff-ILA counts against London-ILI counts for ages 15–64 demonstrating an overall strong (r = 0.64) and statistically significant (P < 0.001) correlation between the two datasets (using log_10_ transformed counts and a permutation test).

Hospital staff-ILA, London-ILI and inpatient-PITR demonstrated similarities in surveillance patterns, with clear seasonal trends similar to those observed with London-ILI data among 15–64 year olds (Figure 1; also see Additional file 1: Figure S1 for interactive surveillance figure), and elevated peaks and troughs of illness at similar time points, but differing magnitudes. With respect to the summer of 2009 (Figure 1), London-ILI (peak rate = 196.8/100,000) demonstrated the burden of influenza to be greater than during any other standard winter influenza season (peak rate range: 11.8-73.0 per 100,000). In contrast to this, hospital staff-ILA estimated the burden of influenza in the summer of 2009 (peak rate = 1142.6/100,000) to be lower than in all winter influenza seasons (peak rate range: 1389.5-2878.2 per 100,000). The number of hospital staff-ILA and London-ILI cases per week across the 5 years were correlated (Figure 1 inset, log_10_ transform, correlation r = 0.64, permutation P < 0.001). This correlation was robust to the age group of ILI used for comparison, with the exception of the very young (<1 year old) and the elderly (>75 years old) (Additional file 2: Figure S2). The similarity of hospital staff-ILA and London-ILI was further demonstrated by calculation of cross-correlation between the time series for different time lags; across seasons, the correlation was generally strongest for a time lag of zero (Additional file 3: Figure S3).

In time series analyses, both hospital staff-ILA and London-ILI showed similar timing of the seasonal component (Additional file 4: Figure S4), although seasonal changes were of lower amplitude in the London-ILI data. There were a greater average number of hospital staff-ILA cases than London-ILI, potentially demonstrating the broader detection of more mild cases among hospital staff.

### Early warning

Whilst cross-correlation and time series analysis demonstrated the similarity of hospital staff-ILA and London-ILI over both high and low rates of symptom reporting, it is the dynamics of the data when symptom reporting is low that is important for providing early warning of increased cases. We examined early warning in two ways; firstly, we used a Bayesian alarm model to examine weekly alarm triggers that indicated weekly increases in cases regardless of the current case rate, and secondly, we used the Moving Epidemic Method to examine seasonal alarms by cases exceeding a seasonally established threshold.

#### Bayesian alarm model

**Figure 2:**
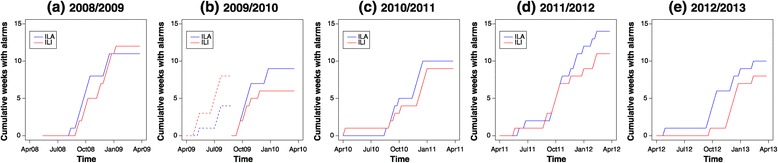
**Cumulative number of weeks with alarms over year-long periods (week 14 in one year to week 13 in the next), for the periods (a) 2008/9, (b) 2009/10 – split between the first (dotted lines) and second (solid lines) parts of the year to show the pH1N1 introduction in the summer of 2009, (c) 2010/11, (d) 2011/12 and (e) 2012/13 for hospital staff-ILA (blue) and London-ILI (red).** Alarms are generated using a Bayesian approach, taking the previous 6 weeks as reference. With the exception of pH1N1 introduction in the summer of 2009, hospital staff-ILA generates alarms at the same or earlier times than London-ILI; however, this is not generally associated with a higher number of alarms (i.e. false positive alarms) over the course of the year.

**Figure 3:**
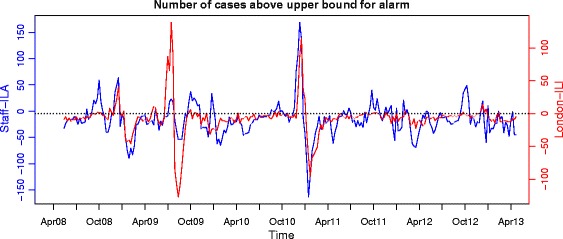
**The number of cases of hospital staff-ILA (blue) and London-ILI (red) above the upper bound for setting off an alarm, by week from April 2008 through April 2013.** Points at or above the line y = 0 represent weeks when an alarm was triggered. The upper bound was set as the 95% quantile of the posterior predictive distribution, based on a baseline rate estimated using the previous six weeks of counts.

We examined the timing of increases in numbers of hospital staff-ILA and London-ILI, based on the observed number meeting or exceeding an upper bound calculated from reference values over the previous 6 weeks, i.e. using a threshold that changes over time. As this approach treats each week independently, and gives an indication of increasing numbers, regardless of the absolute case burden, it is possible to have multiple alarms in a given influenza season. While multiple alarms may be of secondary interest in early warning, they serve as a convenient measure to compare when numbers of hospital staff-ILA and London-ILI increase. Alarms clearly marked increasing numbers of cases, but did not always occur consistently week after week (Figure 1). Hospital staff-ILA demonstrated earlier alarms and greater continuous ‘runs’ in alarms up to peak week than London-ILI. These patterns were robust to the number of previous weeks used as a reference for generating alarms (data not shown). Both London-ILI and hospital staff-ILA data generated a similar number of alarms across the five years, i.e. hospital staff-ILA data were not associated with increased overall alarm rate (Figure 2). Cumulative alarm rates demonstrated a lead time in hospital staff-ILA starting in October of each year. However, in the summer of 2009, when pH1N1 was introduced in the UK, ILI clearly led alarms. To further investigate how alarms were being triggered, we calculated the number of cases above the upper bound for setting off an alarm (Figure 3). Not only were alarms triggered earlier for hospital staff-ILA, but the absolute number of cases above the upper bound was higher for than for London-ILI, suggesting a wider dynamic range. An exception to this is evident in the summer of 2009, when pH1N1 was introduced to the UK.

#### Moving epidemic method

**Figure 4:**
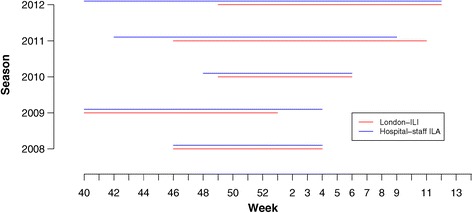
**Estimates of the duration of the epidemic period using the Moving Epidemic Method, for hospital staff-ILA (blue) and London-ILI (red), for influenza seasons (weeks 40 to 20) from 2008/2009 to 2012/2013.** The start and end times of the epidemic period are similar for ILA and ILI, with the exception of the 2012/2013 season, which lacked the characteristic peak of cases typically seen in seasonal influenza.

We used the Moving Epidemic Method to estimate an absolute threshold for hospital staff-ILA and London-ILI for each season (weeks 40–20, i.e., October through March). Although similar fixed thresholds are used for ILI in the UK, no such threshold exists for ILA; the MEM approach, although not used in the UK for surveillance, allowed us to generate comparable estimates of the beginning and end of the epidemic period. Hospital staff-ILA reached the threshold earlier in the 2010/2011, 2011/2012, and 2012/2013 seasons than ILI and at the same time in the two earlier seasons (Figure 4). The difference between the MEM results for ILA and ILI is greatest in the 2012/2013 season; this is due to the unusual dynamics in this season, which lack the characteristic sharp peak of cases typically associated with seasonal influenza (Figure 1).

### Burden of disease

**Figure 5:**
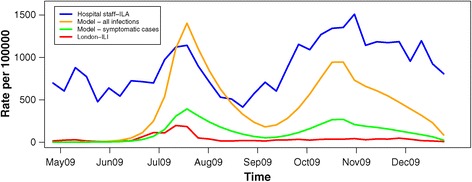
**Rates of London-ILI (red), hospital staff-ILA (blue), estimated true symptomatic cases (green) from Bayesian model, and estimated true asymptomatic and symptomatic infections (yellow) from Bayesian model for April 2009 through January 2010.** Model estimates from Birrell et al. [1].

To determine how well the systems captured burden of disease, we used the introduction of pH1N1 in a natural experimental setting, where the UK was known to have artificially elevated rates of primary-care presentations in summer 2009 and decreased rates in winter 2009/2010 [1,11]. Using this setting, we compared the rates of hospital staff-ILA and London-ILI with estimates of London influenza cases from a mathematical model developed by Birrell et al. [1], which adjusted ILI for levels of healthcare seeking behaviour using data on consultation rates (Figure 5). Similar to the adjusted ‘true’ estimates, hospital staff-ILA demonstrated a larger peak in winter 2009/2010 than in summer 2009, whereas the converse was true for the unadjusted London-ILI, indicating that ILA is likely to be more robust to presentation bias, and hence a better measure of burden of influenza, than primary care presentations.

## Discussion

Hospital staff-ILA data demonstrated seasonal trends in influenza comparable to ILI, with potentially earlier warning and measures of disease burden that were less sensitive to community behaviours, when compared to ILI presentations in the same region. Hospital staff-ILA also provides information on both anticipated surges in patient visits and absences in staff, which are critical in ensuring patient safety in tertiary care settings. If incorporated into existing national surveillance systems, hospital staff-ILA could support annual comparisons of burden of disease and earlier warning. On the local level, this information has the potential for earlier warning for hospitals to prepare their workforce. With appropriate application, this novel surveillance system has the potential to alleviate gaps in current national influenza surveillance highlighted by the WHO [31] and enhance hospital preparedness.

While primary-care ILI surveillance is a sustainable and longstanding system for estimating influenza dynamics, it is susceptible to presentation bias, particularly under certain scenarios e.g. pH1N1, which can affect the estimated disease burden [1,11] and reduce comparability between seasons and countries. The natural experiment that occurred in the UK with the pH1N1 introduction, where presentation bias was estimated through additional studies and presentation was known to be too high in the summer months and too low in the winter months of 2009, provided an opportunity to test the ability for the hospital staff-ILA system to predict burden of disease. Our data showed that hospital-staff ILA was unaffected by this presentation trend and it provided more similar estimates of ‘true’ disease burden, demonstrating that it is robust against such biases. While the UK may have been unique in this ‘natural experiment’, it was not unique with respect to greater pH1N1 disease burden in the winter of 2009/2010 than the summer, which has been observed amongst hospital staff in Hong Kong where intensive surveillance based on confirmatory testing occurred [32]. While modelling different data sources is an effective and common method for dealing with presentation bias, hospital staff-ILA has the potential to provide an additional and currently overlooked data source for this modelling, as well as provide a more consistent, rapid assessment to estimate disease burden in very early response and in particular gives hospitals more accurate and more advanced warning. public health practitioners in Europe have trialled a number of promising surveillance methods, such as the Moving Epidemic Method (MEM) [27] to allow greater comparability between countries and Bayesian synthesis models to better capture true burden [1] and severity [10] of illness. Hospital staff-ILA could support these efforts through providing information to enhance ‘real-time’ estimates of burden, which could be utilised within and between countries in MEM and Bayesian models. Additionally, it may also provide information on a broader range of illness severity, through assessment of number of days of absence. This has the potential to save resources in situations such as pH1N1 where the disease is generally mild, but it is a new introduction.

We observed a clear lead-time in increases in hospital staff-ILA relative to London-ILI starting from October (Figures 3 and 4), which was not associated with an increase false positive signal in staff-ILA (Figure 2). We believe that this trend in lead-time is real, as any general working population will first report absent to work and following this, will present to a healthcare setting. At the local level, earlier lead time would result in a greater ability for hospitals to prepare, which could provide organisational resilience and increase patient safety. Suboptimal nurse-to-patient ratios have been shown to increase patient mortality and adverse events [4,5,33], and could be defined as low organisational resilience when an acute cause, such as an epidemic, results in such a problem. Ideally, hospitals should not just maintain functionality under acute strain or shock (i.e., demonstrate resilience), but rather be able to adapt to and provide for increased demand; early warning, combined with a preparedness plan would help facilitate this. However, NHS hospitals currently rely on traditional surveillance systems for early warning, which have a delay that would not be incurred if surveillance was embedded in the healthcare system. While the use of other data sources for ILI surveillance, such as social media [16,17] and school absenteeism [20,21] has also been shown to increase lead time in syndromic surveillance, causes of peaks in social media and reasons for absenteeism in schools have yet to be fully understood. While our system of hospital staff-ILA requires additional testing before fully incorporating into a national surveillance system, it is clear that earlier prediction of clinical staff absence through hospital-ILA surveillance would enable hospitals to prepare for appropriate staffing and skill mix.

In addition to social media and school-based absentee reporting, a number of additional innovative ILI sentinel sources have been explored, including pharmaceutical sales, emergency department visits, and health helpline calls [15], however, these suffer from presentation bias that is not inherent in hospital-ILA monitoring. While hospital staff-ILA may incur its own set of biases, for example issues related to attending work when ill (‘presenteeism’), which has been predominately observed amongst medical doctors [34-36], these will be different from presentation bias and are likely to be measurable, and therefore hospital staff-ILA will provide a source of data that may counter balance presentation biases. General work absences for ILI surveillance have been explored previously [37], however, focusing on hospital staff is likely to be more effective than other workforces as it is easily embedded in operational management, as observed in Hong Kong [32]. It is also likely to be sustainable and inexpensive as it would be embedded into standard HR procedure, given that it would be tied to important financial activity (i.e., staff payment). Additionally, surveillance of hospital staff-ILA would provide a large, diverse sample with a known denominator. For example, in the UK the NHS has been estimated to be the fifth largest workforce in the world, employing approximately 1.7 million and representing about 2% of the country’s population [38].

While the proposed system has many advantages, there are also some limitations. The age range of hospital staff (20–68 years in our cohort) is not representative of the entire population. However, we found high correlations between hospital-ILA and London-ILI, regardless of age group with the exception of those younger than 1 year-old and older than 74 years, indicating that alternative surveillance for infants and elderly is necessary. For hospital staff-ILA surveillance to be effective, complete cooperation and regular reporting would have to be maintained, and therefore somewhat standardised between organisations. Costs, feasibility and comparability of a larger NHS wide or NHS sentinel site system, would also have to be explored. In settings similar to the ICHT, where physicians may be paid by another entity, i.e., the University, HR does not always obtain a record of absence. However, in our study, exclusion of these staff did not affect our ability to detect influenza, possibly because they represent a small portion of hospital staff. Additionally, daily, electronic reporting of staff illness, reason and type, would have to be supported in order to get the maximum benefit from the system. Currently, at ICHT many departments, especially patient facing clinical services report absences in real-time; while, other departments, may only upload their absences on the system on a weekly basis. However, during the initial months of the H1N1 pandemic, ICHT reported staff absence daily without difficulty, suggesting that with small changes real-time reporting would be possible. For most larger NHS Trusts, staff absences are directly reported into electronic systems designed to capture this information, which would further enable real-time reporting. There are likely to be behavioural factors with respect to taking illness absence that could differ between countries, and these would require further exploration before direct comparisons could be made. Presenteeism, or presenting to work when ill, should be well understood in order to understand biases within the data. Unfortunately, there were not enough cases of inpatient influenza reported in these time periods to examine correlation between inpatient confirmed cases and syndromic hospital staff-ILA cases. Lastly, our data included combined cold, cough and influenza absences, from nationally standardized categories, which were unavoidably combined due to the addition of “influenza” combined with cough and cold after 2009. These are less specific than traditional ILI symptoms and could include other viral respiratory infections; however, even using ILI definitions, it is difficult to differentiate between influenza and other seasonal respiratory viruses based on symptoms alone. Given the similarities in surveillance patterns between ILI and hospital-ILA, it is highly likely that both systems are measuring the same syndromes. Additionally, even if influenza is not the primary cause of the ILA, a surge in viral respiratory illness that renders people unable to work is important to monitor and will have significant public health and hospital resilience implications regardless of the viral pathogen. If a hospital-ILA surveillance system were to be adopted, we would recommend improving standardized illness categories and reporting of symptoms, as well as, initiating voluntary confirmatory testing of respiratory illnesses among hospital staff, which was accepted by HCWs in Hong Kong [32] and is likely to be adopted by a portion of HCWs in the UK.

## Conclusions

While there has been significant focus on how to handle pandemic extremes [39], improving monitoring and planning for seasonal influenza is of great importance [31]. At the community-level, hospital-ILA surveillance has the potential to allow hospitals to better prepare for severe influenza cases and staffing shortage with some early warning. Nationally, hospital-ILA surveillance has potential for earlier warning and to enhance our estimates of influenza burden and severity year-to-year. It may also contribute to rapid and cost efficient syndromic vaccine effectiveness studies, such as those described by Eames and colleagues [40]. We recommend that healthcare and public health organisation investigate the utility of this novel, new source of surveillance, as supplementing current surveillance with hospital-ILA may be an ideal method for addressing sensitivities of current ILI systems [1,11,31] and increasing patient safety.

## Additional files

Additional file 1: Figure S1. Weekly counts of hospital-ILA (blue) and London-ILI in the community (red) from April 2008 to April 2013 (interactive figure). Please use your mouse to ‘zoom in’, change plot type and view numbers of cases. Additional file 2: Figure S2. Scatterplot matrix illustrating correlations between hospital staff-ILA and different age ranges for London-ILI. The lower diagonal panels show a scatterplot for pairs of variables, and the panels on the upper diagonal illustrate Spearman’s rank correlation for each pair of variables. Reading across the top row shows that the correlation between hospital staff-ILA and London-ILI is greatest for ILI in 15-64 year olds, consistent with the age range of the hospital staff, while it is weakest for the very young (<1 year old) and elderly (>75 years old); the first column represents this pictorially. Adjacent correlations on the diagonal shows correlations between age groups within London-ILI showing that the correlation between different age groups in London-ILI is not that different from hospital staff-ILA compared to each age group in London-ILI. Additional file 3: Figure S3. The cross-correlation of hospital staff ILA and London ILI (log_10_ rates) by season. Correlations were calculated between ILA at time t+lag and ILI at time t, and demonstrate that the correlation is generally highest for a time lag of zero weeks. Additional file 4: Figure S4. (a) Rates of hospital staff ILA and London ILI per 100,000 over time, and a time series decomposition of these rates into (b) trend, (c) seasonal and (d) remainder components, using STL decomposition (see Supplementary Methods). Although the overall rate of ILA is higher than ILI (as seen in the trend), the timing of increases and decreases in rate is extremely similar for both seasonal and remainder components.

## Abbreviations

ILI: Influenza-like illness
ILA: Influenza-like absences
pH1N1: Pandemic influenza H1N1 2009
WHO: World Health Organisation
UK: United Kingdom
HR: Human resources
ICHT: Imperial College Healthcare NHS Trust
RCGP: Royal College of General Practitioners
SHA: Strategic Health Authority
