# Supplementary material for: Assessing the use of hospital staff influenza-like absence (ILA) for enhancing hospital preparedness and national surveillance
Source: BMC Infect Dis. 2015 Mar 1;15:110. doi: 10.1186/s12879-015-0789-z (PMC4381490; doi:10.1186/s12879-015-0789-z)
Supplement: Additional file 1: Figure S1. — Weekly counts of hospital-ILA (blue) and London-ILI in the community (red) from April 2008 to April 2013 (interactive figure). Please use your mouse to ‘zoom in’, change plot type and view numbers of cases. [file 12879_2015_789_MOESM1_ESM.docx]

**Additional methods**

Identifying the beginning of outbreaks

We employed a Bayes subsystem [1], using reference values from the previous six weeks due to relatively short, heterogeneous time series. This approach uses the reference values to estimate the rate, assuming that the rate is Poisson distributed with parameter λ. Assuming Jeffreys prior for the rate parameter, λ~Ga(0.5,0), the posterior predictive distribution of the number of cases is distributed according to a negative binomial distribution. An upper bound for the number of cases is calculated based on this distribution (we used the 0.95 quantile), and an alarm is raised if the number of cases is the same or exceeds this upper bound. We modified the estimation of λ from the original study to take into account potentially fluctuating population sizes, by including an offset into the model.

Time series analysis of hospital staff-ILA and London-ILA

Weekly count data were analysed using univariate time series approaches. To compare similarities in hospital staff-ILA and London-ILA, count data were converted to rates per 100,000 and were log_10_ transformed to stabilise the variance. The resulting time series were decomposed into trend, seasonal, and remainder components using seasonal decomposition using loess [2].

**References**

1) Höhle M, Riebler A. The R-Package 'surveillance'. SFB386 Discussion Paper 422. Department of Statistics, University of Munich; **2005**.

2) Cleveland RB, Cleveland WS, McRae JE, Terpenning, I. A Seasonal-Trend Decomposition Procedure Based on Loess. J Official Statist, **1990**;6:3-73.

**Additional figure legends**

Additional Figure 1: Weekly counts of hospital-ILA (blue) and London-ILI in the community (red) from April 2008 to April 2013 (interactive figure). Please use your mouse to ‘zoom in’, change plot type and view numbers of cases.

Additional Figure 2: Scatterplot matrix illustrating correlations between hospital staff-ILA and different age ranges for London-ILI. The lower diagonal panels show a scatterplot for pairs of variables, and the panels on the upper diagonal illustrate Spearman’s rank correlation for each pair of variables. Reading across the top row shows that the correlation between hospital staff-ILA and London-ILI is greatest for ILI in 15-64 year olds, consistent with the age range of the hospital staff, while it is weakest for the very young (<1 year old) and elderly (>75 years old); the first column represents this pictorially. Adjacent correlations on the diagonal shows correlations between age groups within London-ILI showing that the correlation between different age groups in London-ILI is not that different from hospital staff-ILA compared to each age group in London-ILI.

Additional Figure 3: The cross-correlation of hospital staff ILA and London ILI (log_10_ rates) by season. Correlations were calculated between ILA at time t+lag and ILI at time t, and demonstrate that the correlation is generally highest for a time lag of zero weeks.

Additional Figure 4: (a) Rates of hospital staff ILA and London ILI per 100,000 over time, and a time series decomposition of these rates into (b) trend, (c) seasonal and (d) remainder components, using STL decomposition (see Supplementary Methods). Although the overall rate of ILA is higher than ILI (as seen in the trend), the timing of increases and decreases in rate is extremely similar for both seasonal and remainder components.
